# Provision of information, secondary stroke prevention and holistic care for post-transient ischemic attack patients: A scoping review

**DOI:** 10.1097/MD.0000000000042003

**Published:** 2025-05-16

**Authors:** Wei Sheng Ho, Nor Azlin Mohd Nordin, Aznida Firzah Abdul Aziz

**Affiliations:** a Center for Rehabilitation and Special Needs Studies, Faculty of Health Sciences, Universiti Kebangsaan Malaysia, Kuala Lumpur, Malaysia; b Department of Family Medicine, Faculty of Medicine, Universiti Kebangsaan Malaysia Medical Centre, Cheras, Malaysia.

**Keywords:** care, minor stroke, quality of life, residual impairments, transient ischemic attack

## Abstract

Epidemiological evidence suggests that following a Transient Ischemic Attack (TIA), there are residual impairments which threaten quality of life. Despite these consequences, studies addressing post-TIA care remain limited. TIA patients’ needs were summarized into 3 domains: Information, Stroke prevention, and Holistic care. Therefore, this scoping review will evaluate the existing reported care programs with the aim of identifying the scope of the care and its adequacy in fulfilling the patients’ needs. Based on a question “Do available post-TIA care programs sufficiently cover the needs for information, stroke prevention and holistic care?” and using keywords related to TIA and care, this scoping review was conducted in accordance with an established 5-step framework. Searched databases included Scopus, PubMed, Cochrane Library, and EBSCOhost to retrieve potentially relevant studies. Sixteen studies were selected from 1003 relevant articles published between the years 2014 and 2022. We found that all 16 studies included stroke secondary prevention, involving medical treatment, advice on lifestyle changes and exercises, and counseling to post-TIA patients. However, only 4 studies included education and information sharing about post-TIA impairments, while only one study reported the effects of the care on post-TIA impairment namely fatigue. We found no studies which looked into the care impacts on the patients’ overall quality of life. There is a gap in the existing literature regarding holistic care for post-TIA patients, with the management of residual impairments being missed. Further studies targeting a holistic management approach for post-TIA patients are warranted.

## 1. Introduction

Transient ischemic attack (TIA), referred to as “a transient episode of neurologic dysfunction due to focal brain, spinal cord, or retinal ischemia without acute infarction or tissue injury,”^[1]^ is a serious warning for an impending ischemic stroke.^[2]^ The risk of stroke is highest in the first 48 hours following a TIA event, while the risk ranges between 2% and 17% within the first 90 days post-TIA. Because stroke has been a burden to many countries worldwide, including Malaysia, and this disease is the third leading cause of death in the country, prevention of the occurrence of stroke following TIA is crucial. Studies reported nearly 50,000 incident cases with 19,928 deaths, 443,995 prevalent cases, and 512,726 disability-adjusted life years lost due to stroke in Malaysia in 2019 and the number is expected to rise in the near future.^[3]^

In addition to the risk of progression to stroke, it was recently reported that despite symptoms typically lasting less than an hour, more often minutes, a growing body of epidemiological evidence suggests that there is residual impairments post-TIA. These impairments were presented in all aspects of functioning namely cognitive, physical, emotional and psychosocial, which threatened the quality of life of post-TIA individuals.^[4]^ Systematic reviews from 2014 to 2021 documented residual impairments such as depression, cognitive impairment, fatigue,^[5]^ impaired gait,^[6]^ impaired balance,^[7]^ limb paralysis, loss of coordination,^[8]^ and sleep disorder.^[9]^ Aside from that, systematic reviews by other past researchers revealed 15% of a sample of TIA and minor stroke patients were disabled at 90 days as shown with a modified Rankin Scale (mRS) score ≥ 2, with more than 55% of them not having any recurrent events.^[10]^ Another study highlighted cognitive impairments and depression as two important residual impairments that require attention,^[5]^ and without addressing them, the current management is unlikely to be adequate.^[11]^

The current healthcare pathway for post-TIA individuals was said to focus on preventing another stroke,^[12]^ and the same goes with the known existing post-TIA guidelines, in which the residual impairments were not being addressed. A study from 2019 suggested that TIA patients’ needs are multi-components and can be summarized into 3 domains, namely: the need for information covering TIA diagnosis and subsequent stroke risk; secondary stroke prevention; and holistic care, where TIA residual impairments and the impacts on their lives are being addressed.^[13]^ However, despite the concept being proposed 4 years ago, recent paper reported that there is still no guideline that incorporates the treatment of residual impairments as a part of the intervention program to enable the patients to resume their usual daily activities.^[14]^ This group of patients are assumed, often incorrectly, to have made a full recovery.^[15]^ A qualitative study investigating the long-term impact and experiences of follow-up care, found patients reported mixed experiences of follow-up care but largely felt abandoned and alone post-discharge. This marks the existence of discrepancy between the healthcare provider’s beliefs and the patient’s actual needs in the current health pathway. Therefore, this scoping review will evaluate the reported care programs, treatments, and follow-ups for TIA patients with the aim of identifying the scope of programs, their effect and determining if they fulfill the patients’ multiple needs. The findings from this review can be used to guide the planning of further researches and development of a multi-component evidence-based holistic care for individuals with TIA.

### 1.1. Conceptual framework

**Figure 1.**
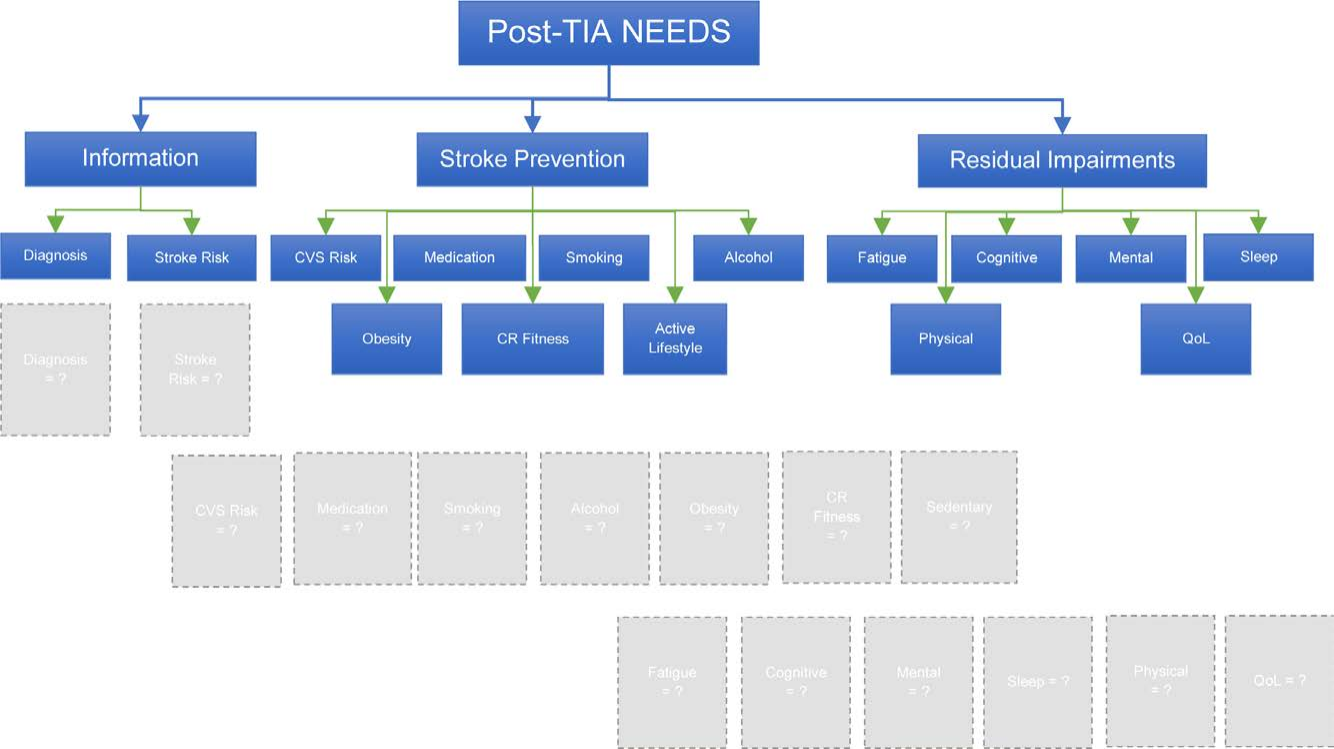
Conceptual framework for the scoping review.

Literature highlighted that post-TIA individuals’ needs cover 3 major domains, namely information, stroke prevention strategies, and holistic approach.^[13]^ This led to our scoping review question: “Do available post-TIA care programs sufficiently cover the needs for information, stroke prevention and holistic care?.” Considering that each domain comprises multi-components, the content of identified care programs will be reviewed and categorized under each component of needs that they are addressing, as illustrated in the conceptual framework in Figure 1.

## 2. Methods

The scoping review was conducted using a 5-step framework, excluding optional step 6 (consultation exercise), suggested by Arksey & Malley,^[16]^ and is reported with reference to the Preferred Reporting Items for Systematic Reviews and Meta-Analyses extension for Scoping Reviews (ScR) Checklist.^[17]^ The protocol of this scoping review has been registered in the Open Science Framework (registration DOI: https://doi.org/10.17605/OSF.IO/BHW2D). This review does not seek to answer efficacy questions but to only map the available literature and the types of care on the topic, identifying key foci, key findings, and the structure of the programs. Further, because this study was a literature review and there was no contact with human subjects, ethical approval was not required.

### 2.1. Eligibility criteria of the targeted studies

Studies were included if they met the following criteria:

1. published in the recent 10 years to capture the most relevant research and programs developed for the topic.
2. availability of full text in English language.
3. provided detailed descriptions of interventions/programs/care for post-TIA individuals.
4. included outcomes relating stroke knowledge, cognition, mood, physical activity (endurance, strength, balance, coordination), adherence, quality of life, sleep quality, level of energy or fatigue, participation, and self-management.

Because this scoping review did not intend to evaluate interventions’ effectiveness, all types of studies were considered in our selection criteria. Excluded were all types of reviews, protocol papers, conference proceedings or abstracts, and guidelines or intervention related to medications or medical procedures only.

### 2.2. Data sources and search strategy

The search terms used are refined through initial searches in PubMed, EBSCOhost, Cochrane Library, and Scopus databases. Keywords in the titles and abstracts of relevant articles were noted, Medical Subject Headings (MeSH) terms were reviewed, and ongoing changes were made to the search strategy until the final terms to be used in the search strategy were deemed sufficient. The keywords were searched in combination with appropriate Boolean operators with parenthesis in the above-listed databases for more accurate results. Truncations were used whenever appropriate. Case-related keywords were saved in the search manager in combination with “TIA” OR “Transient Ischemic Attack” OR “Minor Stroke” OR “Mini Stroke” OR “Non-Disabling Stroke” as search result 1. Meanwhile, care-related keywords are saved as search result 2 in this combination: “Care” OR “Program” OR “Exercise” OR “Rehab” OR “Prevention.” Both search results were then searched using “Search Result 1” AND “Search Result 2.” To narrow down to more relevant results, extra filters of “English language” and “Recent 10 years” are used. The last search was carried out on August 2, 2023; no gray literature was included in the search.

### 2.3. Screening procedure

The search results from all 4 databases were downloaded and imported into EndNote 21.0. Subsequent screenings were executed by the main researcher and verified by a co-researcher. First, auto deduplication was done removing the identified duplicated studies. Since single search strategy through auto-searching to identify duplicates is inadequate, especially type-II duplicates,^[18]^ manual deduplication was supplemented subsequently to remove the unidentified duplicates. Title screening was carried out utilizing advanced search function on Endnote using keywords with Boolean operators used in the database search earlier. Next, abstracts are then being screened through advanced search functions. Collectively, the titles and abstracts of the remaining articles were being screened through manually and studies with title and abstract irrelevant to the case (TIA) and care intended were removed. All types of review, protocol papers and unrelated types of care or studies are being eliminated. Later, the remaining articles undergone full text review and irrelevant studies were then removed at this stage.

### 2.4. Data extraction and charting

Relevant data were extracted from each article by the main researcher who was the main reviewer. An independent reviewer was also involved as a check and balance. The data of interest included authors details, year of publication, research design, sample size and details, interventions used and content details, key findings or outcomes, and study limitations. The extracted data were categorized according to the types of needs by the 2 reviewers who then compared their input and discussed to obtain consensus, and finally tabulated into a summary chart in Microsoft Word version 2409.

### 2.5. Categorization of intervention/care programs

The articles sought were categorized according to the interventional components, and in this case, 4 categories were identified: guideline; education or counseling; mixture of education or counseling and exercise; and exercise. Since physical activity or exercise made a high appearance among the studies in the form of both educational component and structured exercise program, the program is only classified under exercise program (category 3 or 4) if a structured exercise program is prescribed or identified in the study.

The interventions were then further classified according to the targeted need components shown in the conceptual framework. The needs for information described span across the needs for understanding diagnosis and understanding stroke risks.^[13]^ Thus, in this review, studies were classified according to TIA diagnosis and Stroke Literacy, which covers subsequent stroke risks and relevant risk factors.

For the stroke prevention need domain, the interventions were classified if the intervention is relevant to stroke risk factors or stroke prevention. Subgroups available were based on the identified themes across the included articles: blood pressure; blood glucose; lipid or cholesterol; metabolic syndrome; obesity; diet; obstructive sleep apnea; smoking cessation; alcohol consumption; stress; medication adherence; cardiorespiratory fitness; and active lifestyle. Within category 3 or 4, exercise was further specialized into 2 subgroups (cardiorespiratory fitness and active lifestyle) to accommodate exercise programs of different aims: cardiorespiratory fitness or physical capacity (fitness); and to initiate exercise program or exercise adherence (active lifestyle). An intervention program is only considered fit for a “fitness” subgroup if the objective and primary outcome measures are relevant to cardiorespiratory fitness.

Whereas for the holistic care needs domain, in addition to the types of residual impairments (psychological problems, cognitive impairment, fatigue and minor weakness) and impacts of these residual impairments on patients’ lives as described in the qualitative study’s needs categorization,^[13]^ other reported residual impairments by previous studies such as balance, coordination and sleep problem were added. An intervention program was only considered fulfilling this need if: the residual impairment(s) was identified as a sequel after TIA in the article; the objective was relevant to the residual impairment management; and the primary outcome measure was relevant to the residual impairment.

## 3. Results

### 3.1. Search result

**Figure 2.**
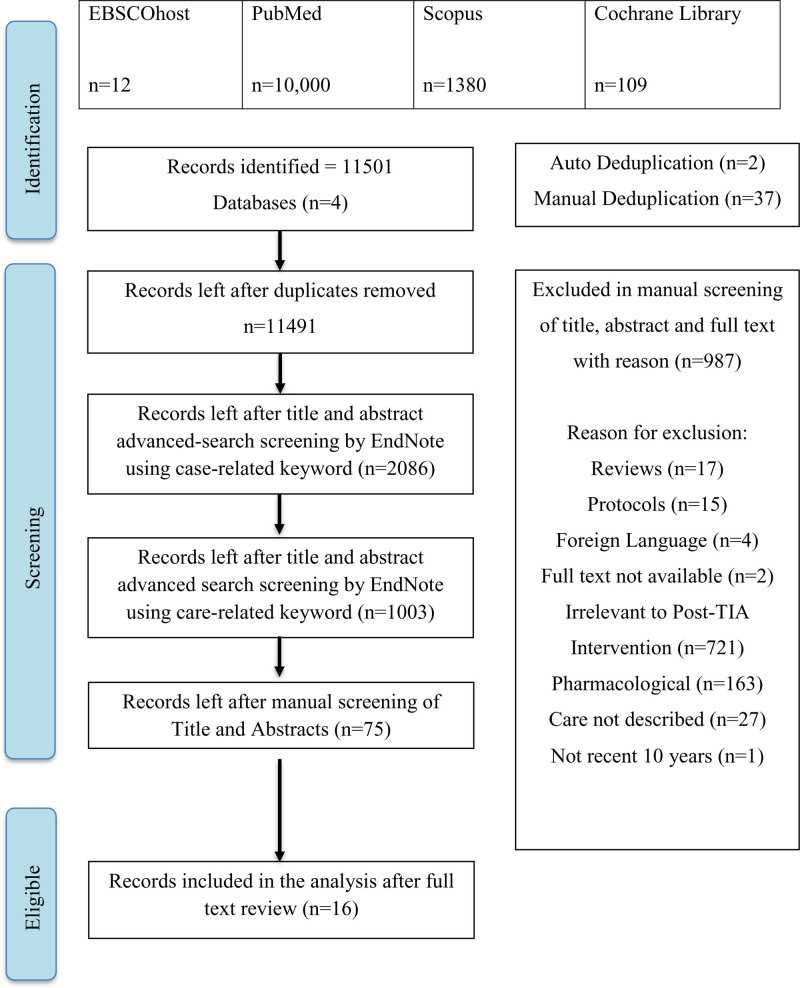
PRISMA flowchart of articles/studies search and selection.

The initial search yielded a total of 11,501 articles from the 4 databases. Title and abstract screening through the advanced search function on EndNote to eliminate studies without case-related keywords has led to a remaining relevant result of 2086 articles. Advanced search to screen titles and abstracts with care-related keywords has further reduced the number to 1003. Following manual screening of the title and abstract, the number was further reduced to 75. From this, 16 studies were finally included after reviewing the full text, of which 15 are original studies, and one is a guideline. The other 59 articles were removed for reasons such as duplicated articles, full text not available, care not described, and full text not available in English. A summary of the screening results was documented in a Preferred Reporting Items for Systematic Reviews and Meta-Analyses flow diagram as shown in Figure 2.

### 3.2. Characteristics of the included studies

The 16 studies included in this review were published between 2014 and 2022 and included one guideline, 9 randomized controlled trials, one qualitative descriptive study, two prospective cohort studies, one retro-cohort study, one iterative development study, and one case study. Sampled populations from these studies were populations with TIA or minor stroke (n = 1996). Reported care programs included exercises and programs for the purpose of lifestyle modification, which encompasses education and information sharing on physical activity, smoking cessation, and lifestyle modification. Specifically, this scoping review identified programs available in the form of guidelines (n = 1), exercise intervention (n = 2), educational or counseling intervention (n = 4), and mixed regimens of exercise with education or counseling intervention (n = 9). Most identified interventions were on a face-to-face basis, while there were two innovations in an attempt to support lifestyle modification through the utilization of mobile applications. Details of the studies included in this review are shown in Table 1 and Table 2. Interventions are also categorized in a summary table (Table 3).

**Table 1:** Key metrics of the selected studies

Author	Study design	Study region	Objective	Population	Outcome measure	Key findings
Kernan et al (2014)^[19]^	Guideline	N/A	To provide comprehensive and timely evidence-based recommendations on the prevention of future stroke among survivors of ischemic stroke or TIA.	Not applicable	Not applicable	Not applicable
Prior et al (2017)^[20]^	Prospective Cohort Trial	United Kingdom	Describe subacute postTIA/MNDS psychological outcomes; and test our hypothesis that CCR, associated with risk factor reduction post-TIA/MNDS, 2 would be independently associated with improved anxiety, depression, QoL, and cognition.	Patients with ≥ 1 risk factor, within 11.5 weeks post-TIA/MNDS (n = 80)	- HADS - SF-12	Anxiety and executive dysfunction persisted post-TIA/MNDS. CCR was not independently associated with psychological improvements. CCR psychological treatment may benefit depression.
Sajatovic et al (2018)^[21]^	Randomized Controlled Trial	United States	Evaluate effects of a novel behavioral TargetEd MAnageMent Intervention (TEAM) versus treatment as usual (TAU)	Age < 65 who had a stroke or transient ischemic attack and a Barthel index score of > 60 (n = 38)	- Blood pressure - HbA1c - Lipids - Medication adherence - Weight - Health behaviors (diet, exercise, smoking, substances), - Depression - Quality of life - Perspectives of TEAM participants.	TEAM participants had significantly lower mean systolic blood pressure by 24 weeks, improved HbA1c and high-density lipoprotein cholesterol (*P* = .03). Other biomarkers and health behaviors were similar between groups. Qualitative results suggested improved awareness of risk factors as well as positive effects of group support.
Irewall et al (2019)^[22]^	Randomized Controlled Trial – Sub Study	Sweden	To analyze the impact of 2 forms of secondary preventive follow up on the association between education level and levels of blood pressure (BP) & low-density lipoprotein cholesterol (LDL-C) after stroke/TIA.	Stroke and TIA patients (n = 771)	- Blood pressure	Nurse-led, telephone-based secondary preventive follow-up led to comparable improvements in BP across education groups, while routine follow-up disfavored those with low education.
Heron et al (2021)^[23]^	Iterative Development	United Kingdom	Explore perceptions on usability and relevance of the app in order to maximize user engagement and sustainability.	Scoping review (n = 29) FGD (n = 32)	- Perceptions on usability and relevance of the Brain Fit App	Not applicable
Patomella et al (2021)^[24]^	Case study	Sweden	To investigate the feasibility and acceptability of a digitally supported lifestyle program called “Make My Day” (MMD) for people at risk for stroke following a transient ischemic attack, and (2) to describe participants’ stroke risk and lifestyle habits pre- and post-intervention.	Participants at risk of stroke (n = 6)	- Feasibility - Stroke Risk card - Swedish Lifestyle Habits Survey - EQ-5D (QoL) - Global Rating of Change Scale	MMD program was highly accepted by the participants including the interventionists & primary healthcare professionals, that lifestyle change goals could be achieved, and that health could be improved. Physical activity goals hard to sustain over time, but dietary goals were maintained. The mHealth service used in the program (mobile phone app) was accepted and usable for the participants.
Kamm et al (2014)^[25]^	Prospective single-center interventional cohort study	Switzerland	To evaluate the feasibility and effectiveness of a comprehensive outpatient rehabilitation program combining secondary prevention and neurorehabilitation to improve vascular risk factors, neurologic functions, and health-related quality of life (HRQOL) in patients surviving a transient ischemic attack (TIA) or stroke with minor or no residual deficits.	Patients with sustained TIA or stroke with 1 or more vascular risk factors (n = 105)	- 9-Hole Peg Test (9-HPT) - 6-Minute Walk Test (6MWT) - One Leg Stand (OLS) test.	Considerable improvement in vascular risk factors as well as in a substantial improvement in neurologic functions and HRQOL. It was practicable and well accepted and could be easily integrated into an existing CCR.
Morén et al (2016)^[26]^	Randomized Controlled Trial	Sweden	Objectively measure the effect of PaP on physical activity and physical capacity, as well as self-rated health at 3 and 6 months after TIA.	TIA Patients (n = 88)	- Physical Activity - Physical Capacity - Self-rated Health - Body Mass Index (BMI)	PaP did not increase physical activity after TIA; however, there was an increase in physical capacity.
Marzolini et al (2016)^[27]^	Retro Cohort Study	Canada	Determine effects of cardiac rehabilitation (CR) on cardiovascular fitness (peak oxygen uptake [VO2peak]) and 6-minute walk distance (6MWD) post TIA.	Post TIA patients (n = 85)	- Adherence - Anthropometrics - Cardiovascular fitness - Depression	A CRP is feasible and effective for improving cardiovascular health.
Vahlberg et al (2021)^[28]^	Randomized Controlled Trial	Sweden	Investigate whether daily mobile phone text messaging coupled with the use of a training diary & a pedometer were better than current standard treatment to improve body composition & cardiometabolic risk markers & self-reported health at 3 months after stroke.	Intervention (n = 40)	- Fat mass and fat-free mass - Cardiometabolic risk factors like blood lipids, glycated hemoglobin and blood glucose	No clear effect was detected on body composition, cardiovascular biochemical risk factors or self-perceived health.
Vahlberg et al (2020)^[29]^	Randomized Controlled Trial	Sweden	Evaluate a 3-month program consisting of daily mobile phone instructional text messaging, combined with a 2-week use of a pedometer to record walking distance, and the use of a training diary.	Intervention (n = 40)	- 6MWT & chair-rising - Mobility, gait speed, handgrip strength, body composition (fat mass and muscle mass), biochemical risk-markers, HRQoL, and cardiovascular events.	3 months of daily mobile phone text messages with guided training instructions improved composite mobility measures (walking performance & lower body strength)
Sammut et al (2021)^[30]^	Qualitative Descriptive Study	Australia	Explore perceptions of participation in a secondary stroke prevention program (delivered by a community-based multidisciplinary health service team within a community gym) by adults with TIA or mild stroke.	Adults with TIA or Mild Stroke (n = 30)	- Participants’ Perceptions	People with TIA or mild stroke who participated in a community-gym-based health service delivered program perceived value in the accompanying health professional support and being in a group, highlighting the role these features play in changing physical activity behaviors.
Deijle et al (2022)^[31]^	Randomized Controlled Trial	Netherlands	Investigate the effect of a 1-year exercise intervention on cognition in patients after a TIA or minor ischemic stroke.	TIA or stroke patients. Intervention (n = 60)	- Global Cognitive Functioning (MoCA) - Cardiorespiratory Fitness - Cardiovascular Profile - Attainment of secondary prevention targets - Anxiety - Depression - Fatigue	No benefit on global cognitive functioning. Positive effect of intervention on fatigue.
MacKay-Lyons et al (2022)^[32]^	Randomized Controlled Trial	Canada	Investigate short and long-term effects of a community-based program of rehabilitative exercise and education as an adjunct to pharmacological management to reduce vascular risk factors in people after NDS/TIA	Participants < 3 months of NDS or TIA (n = 184)	- Systolic Blood Pressure - Diastolic blood pressure; high-density lipoprotein cholesterol; low-density lipoprotein cholesterol (LDL-C); total cholesterol; triglycerides, fasting glucose, and BMI - Patient-Reported Psychosocial	Modest impact of exercise-induced vascular risk factor reduction for secondary stroke prevention Significant between-group differences found in DBPrest and LDL-C at post-intervention, in favor of the PREVENT group, were not sustained at follow-up.
Hedegaard et al (2014)^[33]^	Randomized Controlled Trial	Denmark	Investigate the effectiveness of a multifaceted intervention including MI in improving medication adherence for secondary stroke prevention.	Patients with acute first-time ischemic stroke or TIA (n = 180)	- Composite medication possession ratio (MPR) for antiplatelets, anticoagulants and statins - Adherence and persistence to specific thrombopreventive medications at 12 months - Combined end point of cardiovascular death, stroke or acute myocardial infarction - Patient satisfaction with the service	A multifaceted pharmacist intervention including MI did not improve adherence or persistence to secondary stroke prevention therapy and had no impact on clinical outcomes. However, due to the high adherence rates, only little room for improvement existed.
McAlister et al (2014)^[34]^	Randomized Controlled Trial	Canada	Evaluate whether a pharmacist case manager could improve risk factors among survivors of stroke or transient ischemic attack.	Ischemic stroke or transient ischemic attack patients (n = 279)	- Blood pressure - Lipid control	Nurse-led case management program based on monthly evaluation of risk factors, patient counseling and feedback to primary care physicians improved control of key risk factors for stroke (hypertension and dyslipidemia) by 6 months. However, even greater improvements were seen among patients whose care was managed by a pharmacist case manager who was empowered to initiate and titrate medications to attain guideline-recommended targets.

CCR = comprehensive cardiac rehabilitation, CRP = cardiac rehabilitation program, HADS = hospital anxiety and depression scale, Hb1AC = glycated hemoglobin, MNDS = mild non-disabling stroke, PaP = physical activity on prescription, QoL = quality of life, SF-12 = 12-item short form survey, TIA = transient ischemic attack.

**Table 2:** Description of interventions reported in the included studies

Author	Types of programs	Professionals involved	Timing	Relevant care program
Kernan et al. (2014)^[19]^	Stroke prevention: -Lifestyle modification	N/A	N/A	Intervention: - Lifestyle modification *Comorbidities: • Hypertension (Salt restrictions; diet rich in fruits & vegetables; low fat dairy products; regular physical activity; limited alcohol consumption) • Dyslipidemia (lifestyle modification, dietary recommendations, and medication recommendations) • Disorders of Glucose Metabolism and DM (Use of existing guidelines from the ADA for glycemic control and cardiovascular risk factor management is recommended for patients with an ischemic stroke or TIA who also have DM or pre-DM) • Overweight and Obesity (usefulness of weight loss among patients with a recent TIA or ischemic stroke and obesity is uncertain) • Metabolic Syndrome (counseling for lifestyle modification (diet, exercise, and weight loss) for vascular risk reduction) *Nutrition: • Counsel patients with a history of stroke or TIA to follow a Mediterranean-type diet instead of a low-fat diet • Reduce sodium intake to less than ≈ 2.4 g/d. Further reduction to < 1.5 g/d is also reasonable and is associated with even greater BP reduction • Patients with a history of ischemic stroke or TIA and signs of undernutrition should be referred for individualized nutritional counseling *Physical inactivity • At least 3 to 4 sessions per week of moderate- to vigorous-intensity aerobic physical exercise, average 40 minutes • Referral to a comprehensive, behaviorally oriented program is reasonable *Smoking Cessation: • Advise every patient with stroke or TIA who has smoked in the past year to quit • Advise patients after TIA or ischemic stroke to avoid environmental (passive) tobacco smoke • Counseling, nicotine products, and oral smoking cessation medications are effective in helping smokers to quit *Alcohol Consumption: • Heavy drinkers should eliminate or reduce their consumption of alcohol • Light to moderate amounts of alcohol consumption (up to 2 drinks per day for men and up to 1 drink per day for nonpregnant women) may be reasonable
Prior et al. (2017)^[20]^	Stroke prevention: -Exercise -Lifestyle modification Holistic: -Exercise: Mental health	Not stated	Not stated	Interventions: Comprehensive Cardiac Rehabilitation (CCR) - Exercise & Lifestyle modification: • Medical and nursing case management • Exercise • Dietary counseling • Smoking cessation • Psychological services.
Sajatovic et al. (2018)^[21]^	Stroke prevention: -Education	1.Nurse educator 2. Peer dyad	Within 12 months of hospital discharge or release from an emergency department.	Intervention: TargetEd ManageMent (TEAM) - Lifestyle modification: • 60-minute initial 1:1 session, nurse educator and peer dyad met with the stroke/TIA survivor for introductions, orientation, and logistic planning. • Group sessions co-led by the nurse educator and peer dyad, using a detailed curriculum with semi formal scripting. • Seven brief (approximately 10–20 minutes) telephone sessions were implemented over the 6 months to reinforce content from the group sessions, served as a behavioral model, provided social support, and facilitated linkage with other care providers (stroke nurse/patients).
Irewall et al. (2019)^[22]^	Stroke prevention: -Education -Lifestyle modification -Medication adherence	Not stated	Not stated	Intervention: - Lifestyle modification: • Follow-up included information about measurement results, lifestyle counseling (physical activity, diet, and smoking cessation), and assessment of pharmacological treatment.
Heron et al. (2021)^[23]^	Information: -Diagnosis Stroke prevention: -Exercise -Goal settings -Lifestyle modifications Holistic: -Management tips: Fatigue	Not stated	Not stated	Intervention: (Brain-Fit App) - Information: • TIA & Minor Stroke • Goal Setting, Action Plan - Lifestyle modification: *Physical Activity: • Information • Recommended types of Exercise • Physical activity diary *Healthy Diet • Recommendations & Setting Targets *Fatigue management: • Hints & other treatments *Smoking Cessation
Patomella et al. (2021)^[24]^	Information: -Stroke literacy Stroke prevention: -Lifestyle modification -Exercise -Goal settings	Interventionist/researcher together with a trained health professional (trained during 2 half-days), i.e., an occupational therapist, a physiotherapist, or a dietician.	Not stated	- Lifestyle modification: (1) Individual meeting which participants set 3 individual goals concerning their needs and motivation. (Goals included regular participation in health-promoting EEAs and change in lifestyle habits, e.g., healthy eating, exercise, and physical activity.) (2) Five face-to-face group sessions over 5 weeks with a booster session 5 weeks later for purpose to create awareness of stroke risk factors and current activity patterns. (3) Between face-to-face sessions, a digital mHealth platform (app) was used by participants to monitor their progress toward their goals, including participation in meaningful EEAs.
Kamm et al. (2014)^[25]^	Information: -Diagnosis -Stroke literacy Stroke prevention: -Lifestyle modification -Exercise Holistic: -Exercise: weakness, coordination, balance	1.Cardiovascular exercise therapists 2. Physiotherapist specialized in neurorehabilitation. 3. Neurologists 4. Cardiologists 5. Nutritionists 6. Neuropsychologists	Not stated	Intervention: - Information: Thursday: 1 hour: • Lecture and counseling (3 lectures on etiology, diagnosis, treatment, and prevention of stroke by neurologists; 1 lecture on vascular risk factors by cardiologists) - Exercise: *Tuesday: First hour: • Alternating aerobic ergometer training/nordic walking given by cardiovascular exercise therapists. • Intensity = 70% and 85% of the peak heart rate of the baseline exercise test. *Thursdays: 45 minutes • Aerobic ergometer training - Lifestyle modification: Thursday:1 hour: • 5 lectures on nutrition counseling by nutritionists; 2 lectures on active and passive smoking cessation; and 1 lecture on psychological coping strategies given by neuropsychologists - Impairments: Tuesday: Second hour: • Defined sessions to address special problems of patients with stroke including endurance training, fine motor skills, coordination, balance training, mobilization, or weight training given by a physiotherapist specialized in neurorehabilitation.
Morén et al. (2016)^[26]^	Stroke prevention: -Exercise -Education	Physical Therapist	Not stated	Intervention: physical activity on prescription (PAP): - Exercise: (1) Motivational interviewing technique was used to identify the most suitable physical activity to prescribe and to help build and strengthen motivation and strategies for change. (2) The prescriptions included intensity, frequency, & duration of the activity prescribed. No target for heart rate was given, but supported to do activity at an intensity level such approximately 12–13 on the RPE scale (warm and breathless).
Marzolini et al. (2016)^[27]^	Stroke prevention: -Exercise -Education	1.Physicians 2. Physiotherapists 3. Nurses 4. Kinesiologists 5. Psychologists 6. Dietitians.	Not stated	Intervention: cardiac rehabilitation - Exercise: • 90-minute exercise classes once per week for 6 months • Exercise classes included aerobic training & resistance training • The initial walking: 1.6 km/day, intensity and/or 60%-80% of peak oxygen uptake (VO2peak), progressed every 2 weeks, increase to max. 6.4 km and then increase intensity to a max. 80% of VO2peak (max. 60 minutes). • Resistance training: 3 lower body, 5 upper body, and 2 trunk-stabilizing exercises. Gradually progress from 10 to 15 repetitions and then to increase resistance by 5 kg or 1 band level and reduce repetitions to 10. - Lifestyle education: • Education sessions, as well as psychosocial and dietary counseling
Vahlberg et al. (2021)^[28]^	Stroke prevention: -Exercise	Not stated *Instructional text messages sent via SMS	Not stated	Intervention group: - Exercise: (1) daily instructional text messages via SMS for 3 months on how to exercise to increase walking performance and improve lower body strength (2) the use of training diaries for 3 months (3) pedometers for the registration of step counts during week 1 and week 12 **Detail: (1) Walk 10 minutes daily at a moderate intensity (12–13 on the Borg exertion scale) for the first 2 weeks, then (2) gradually increase the walking time and the exertion to a perceived strenuous intensity by the third month up to 15 on the Borg exertion scale by performing outdoor walking either as a 30-minute walk or in intervals. (3) Functional lower body exercise: to repeatedly rise from a sitting position without support, repetitions starting from 10 rises to 15 rises repeated 3 times per day. (4) Patients were instructed to rest 1 day per week, that is, being active but not necessarily doing walking exercises
Vahlberg et al. (2020)^[29]^	Stroke prevention: -Exercise	Not stated *Instructional text messages sent via SMS	Not stated	Intervention Group: - Exercise: (1) daily instructional text messages via SMS for 3 months on how to exercise to increase walking performance and improve lower body strength (2) the use of training diaries for 3 months (3) pedometers for the registration of step counts during week 1 and week 12 **Detail: (1) Walk 10 minutes daily at a moderate intensity (12–13 on the Borg exertion scale) for the first 2 weeks, then (2) gradually increase the walking time and the exertion to a perceived strenuous intensity by the third month up to 15 on the Borg exertion scale by performing outdoor walking either as a 30-minute walk or in intervals. (3) Functional lower body exercise: to repeatedly rise from a sitting position without support, repetitions starting from 10 rises to 15 rises repeated 3 times per day. (4) Patients were instructed to rest 1 day per week, that is, being active but not necessarily doing walking exercises
Sammut et al. (2021)^[30]^	Stroke prevention: -Education -Exercise	Health professional	Not stated	Intervention: S + SLAM-TIA (Face to Face Health Professional Led Community Stroke Team; Group based 2 sessions/week for 6 weeks at Community Gym Setting) - Exercise: • Supervised Physical Activity: 60 minutes, progressive tailored aerobic physical activity and resistance weight training; Self progression encouraged; Self Selection of Activities - Lifestyle modification: Education: 30minites (Identifying Stroke; Lifestyle changes including diet, alcohol, tobacco use, stress, sleep, relevance and recognition of MVPA)
Deijle et al. (2022)^[31]^	Holistic: -Exercise: Cognitive impairment	Specialized physiotherapists	Less than 1 month since the onset of signs and symptoms	Intervention: - Exercise: 2 Visits to Outpatient clinic during first 3 months after TIA/Minor Stroke • Current exercise behavior and motivation were assessed and exercise goals were established. • 12-week exercise program in groups of 10 patients; 2× 1-hour sessions of exercise training per week, supervised by TWO specialized physiotherapists • Exercise consists of Aerobic, Strength Training & Home-Based Exercise After the completion of the 12-weeks group exercise program, follow-up consisted of 3 visits to the physiotherapist over a 9-month period for counseling by the physiotherapists, based on motivational interviewing to maintain an active lifestyle and continue exercising.
MacKay-Lyons et al. (2022)^[32]^	Information: -Diagnosis Stroke prevention: -Education -Exercise -Lifestyle modification	1.Physiotherapist 2.Kinesiologist 3.Multidisciplinary team	Within 3 months of first probable or definite TIA	PREVENT Intervention - Exercise: • Meetings to identify personal health goals, and related barriers & facilitators • exercise sessions supervised by health professionals • user-friendly health passports • positive reinforcement, adult learning strategies Discussion on - Lifestyle modification: • Stressed importance of continuing physical activity after completion of the program for 30 minutes per day, 5 days per week at a rating of perceived exertion (RPE) of “somewhat hard” to “hard" was being stressed. Discussion on: 1. Heart healthy eating: the basics, 2. Goal setting, 3. Exercise: The Basics, 4. Cardiovascular risk factors and BP self-monitoring, 5. Nutrition: building on the basics, 6. Exercise: Building on the basics, 7. Cardiovascular medications, 8. Healthy weight, 9. Smoking cessation, 10. Stress and coping, 11. Fine-tuning healthy eating, and 12. Wrap-up.
Hedegaard et al. (2014)^[33]^	Stroke prevention: -Medication adherence -Lifestyle modification	Clinical pharmacist	Within 30 days from diagnosis	(1) Medication Review: Thrombopreventive agents & potential adherence problems related to these. (2) Patient interview (6 Months post-Discharge): face-to-face patient interview to support adherence and lifestyle changes, dialogue based on Motivational Interview. At the end of the interview, the patients received A written summary of the interview including their own goals and a list of jointly agreed-on actions that should be taken. (3) Follow-Up Telephone Calls: 1 week as well as 2 and 6 months after discharge.
McAlister et al. (2014)^[34]^	Stroke prevention: -Medication adherence -Lifestyle modification	Pharmacists Nurses	Not stated	Control: (Nurse Led): 6 months 1. Lifestyle advice (exercise, low-salt diet, smoking cessation, medication adherence) 2. Checked the patient’s blood pressure and LDL level, and faxed blood pressure measurements and a list of current medications to the patient’s primary care physician Intervention: (Pharmacist Led): 6 months 1. Initiated or titrated antihypertensive and/or lipid-lowering therapy as appropriate (using treatment algorithms and targets consistent with current Canadian guidelines). 2. Lifestyle advice

DM = diabetes mellitus, EEA = Engaging Everyday Activities, MVPA = Moderate-to-Vigorous Physical Activity, S+SLAM-TIA = Service change and Supporting Lifestyle and Activity Modification after TIA, SMS = Short Message Service, TIA = transient ischemic attack.

**Table 3:** Intervention components of the included studies based on the targeted categories

Studies	Information		Secondary prevention												Holistic			
	TIA Diagnosis (n = 1)	Stroke Risk/literacy (n = 5)	BP (n = 2)	Cholesterol/lipid (n = 2)	Glucose (n = 1)	Metabolic syndrome (n = 1)	Obesity (n = 2)	Diet (n = 8)	OSA (n = 1)	Smoking cessation (n = 9)	Limit alcohol (n = 3)	Medication adherence (n = 5)	Active lifestyle (n = 10)	CR fitness (n = 3)	Fatigue (n = 1)	Cognitive (n = 1)	Mental (n = 2)	Weakness (n = 1)
Kernan et al (2014)^[19]^			**/**	**/**	**/**	**/**	**/**	**/**	**/**	**/**	**/**		**/**					
Prior et al (2017)^[20]^								**/**		**/**			**/**				**/**	
Sajatovic et al (2018)^[21]^												**/**						
Irewall et al (2019)^[22]^								**/**		**/**			**/**					
Heron et al (2021)^[23]^		**/**								**/**		**/**	**/**		**/**			
Patomella et al (2021)^[24]^		**/**						**/**		**/**			**/**					
Kamm et al (2014)^[25]^	**/**	**/**						**/**		**/**				**/**			**/**	**/**
Morén et al (2016)^[26]^		**/**											**/**					
Marzolini et al (2016)^[27]^								**/**						**/**				
Vahlberg et al (2021)^[28]^													**/**					
Vahlberg et al (2020)^[29]^													**/**					
Sammut et al (2021)^[30]^		**/**								**/**	**/**		**/**					
Deijle et al (2022)^[31]^														**/**		**/**		
MacKay-Lyons et al (2022)^[32]^		**/**	**/**		**/**		**/**	**/**		**/**	**/**	**/**						
Hedegaard et al (2014)^[33]^												**/**						
McAlister et al (2014)^[34]^								**/**		**/**		**/**	**/**					

BP = blood pressure, CR Fitness = cardiorespiratory fitness, OSA = obstructive sleep apnea, TIA = transient ischemic attack.

### 3.3. Review findings

All 16 reviewed studies covered interventions targeting components of secondary stroke prevention, primarily through modifiable risk factors management such as cardiovascular risk factors, diet, medication adherence, physical inactivity, smoking, and alcohol consumption. In general, interventions addressing the need for information and holistic approaches were limited, with 6 and 4 relevant studies, respectively.

#### 3.3.1. Interventions

Out of 16 studies, only 6 documented interventions relevant to the needs of information as part of the program. This need was addressed through several programs namely, Brain Fit App,^[19]^ Cardiovascular and Neurologic Rehabilitation,^[20]^ Program of Rehabilitative Exercise and Education to Avert Vascular Events After Non-Disabling Stroke or Transient Ischemic Attack (PREVENT) program,^[21]^ Service change and Supporting Lifestyle and Activity Modification after TIA (S + SLAM-TIA) program,^[22]^ Make My Day program,^[23]^ and physical activity on prescription (PaP).^[24]^ Topics covered in the program included diagnosis and signs of TIA or stroke, etiology, treatment, vascular risk factors, and lifestyle modification strategies. Information was conveyed through traditional methods such as face-to-face education, lectures, and counseling, as well as through mobile applications such as the Brain Fit App. Face-to-face sessions were conducted individually or in groups, lasting 45 to120 minutes per session, once a week for 6 to12 weeks.

In the context of stroke risk prevention, studies were found focusing on addressing monitoring of various modifiable risk factors such as blood pressure, glucose levels, cholesterol, obesity, smoking and alcohol consumption habits. While guideline by Kernan et al^[25]^ was extensive in nature, classifying suggestions on health targets and relevant approaches according to risk factors and comorbidities, by level of evidence, potential types of supportive programs were not noted. Other interventions identified for this purpose provided supporting information by the means of education sessions, counseling, and the use of mobile apps. Topics covered in these interventions ranged from monitoring blood pressure to promoting healthy dietary habits and physical activity. Face-to-face sessions typically last 45 to 120 minutes per session, once per week, for 6 to12 weeks were implemented. Patients were also referred to therapeutic clinics for further support, such as management of hypertension and diabetes, smoking cessation, and diet and weight loss program. Peer dyads^[26]^ and pharmacists^[27,28]^ were also involved for support and the titration of medications, and one study recorded follow ups by nurses. In addition, a comprehensive mHealth platform^[23]^ was also noted, which offered to track daily activities, smoking habits, stress levels, and dietary intake to empower patients in modifying their lifestyles. Simple empowerment tools like diaries and instructional messages via Short Message Services (SMS)^[29,30]^ was also utilized to encourage exercise and healthy habits.

Two categories of exercise interventions were identified in stroke prevention documented in the studies, namely to promote exercise adherence for sedentary individuals; and to improve cardiorespiratory fitness. In efforts to combat sedentary behavior, education on exercise was provided through nurse and pharmacist-led case management and followed up in 3 studies.^[27,28,31]^ Other strategies identified included Engaging Everyday Activities (EEAs),^[23]^ PaP,^[24]^ mHealth platforms for progress registering and tracking,^[23]^ group-based exercises^[20–23,26,32]^ with an individualized session for aerobic and strength training in the community gymnasium,^[32]^ structured programs prescribed via the app with goal-setting features^[19]^ and daily SMS instructions to perform walking training and raising from a chair without support exercises.^[29,30]^ Nonetheless, the exercise program utilized by Prior et al^[33]^ was not specified with regard to its purpose and components. As such, it was placed under the sub-group of active lifestyle as a mean of exercise initiation.

Regarding cardiorespiratory fitness, 3 studies incorporated aerobic exercises,^[20,32,34]^ with strength training included in two of them.^[20,32]^ Sessions typically lasted 60 to 90 minutes, 2 to 3 times a week, over a period of 3 to 6 months. Participants started with moderate-intensity aerobic exercises of either 40% Target Heart Rate,^[32]^ 70% Peak Heart Rate,^[20]^ 60% Peak Oxygen Consumption (VO2 Peak) or 1.6 km walk per day,^[34]^ gradually increasing to reach higher intensity levels. Strength training involves multiple sets at a percentage of one’s maximum capacity, progressing by weight or resistance level but reducing repetitions. Two of the programs were conducted in group settings.^[20,32]^

In the context of holistic care, only 4 studies included interventional programs targeting residual impairments in post-TIA patients. These impairments included fatigue,^[19]^ cognitive issues,^[32]^ mental health concerns,^[20,33]^ weakness, balance, and coordination,^[20]^ while sleep problems were not being addressed. For instance, Heron et al focused on fatigue management through the Brain Fit App.^[19]^ Deijle et al addressed cognitive impairments with the MoveIT program,^[32]^ while Prior et al tackled mental health with the Comprehensive Cardiac Rehabilitation (CCR) program, offering a combination of exercise, medical management, counseling, and psychological services.^[33]^ Kamm et al developed a holistic program targeting mental health, weakness, balance, and coordination issues.^[20]^ While specifically designed for stroke patients, the model may also benefit TIA patients and serve as a reference for future studies on post-TIA residual impairments.

#### 3.3.2. Guiding concepts, materials and approaches

Various concepts, materials and approaches were applied or utilized as guidance across sixteen studies to engage and motivate patients. These included adherence,^[30]^ self-management,^[22,26]^ motivational interviewing,^[23,24,27,32]^ peer dyads,^[26]^ behavioral analysis,^[19]^ EEAs, and the Canadian Occupational Performance Measure.^[23]^ Motivational interviewing, used in setting goals for lifestyle changes, was the most prevalent approach (n = 4). Sajatovic’s TargetEd MAnageMent Intervention (TEAM) program emphasized patient empowerment through self-management,^[26]^ while Sammut’s S + SLAM-TIA program focused on self-management based on competence, autonomy, and relatedness from self-determination theory.^[22]^ On the other hand, Vahlberg et al employed instructional messages to enhance adherence to exercises, increasing therapy intensity without additional therapist time.^[30]^ The Brain Fit App employed in a study also utilized reminders and motivational messages to promote engagement.^[19]^ Patomella et al integrated EEAs to involve patients in health-promoting activities which they found valuable, with Canadian Occupational Performance Measure-guided motivational interviewing to supplement the program.^[23]^

#### 3.3.3. Timing and professionals involved

Of the 16 studies, we found 4 studies in which the timing of intervention initiation was stated. Generally, the timing of the intervention initiation ranges from as early as 30 days from the diagnosis^[27]^ or onset of signs and symptoms of TIA^[32]^ to within 12 months of hospital discharge or release from an emergency department^[26]^; with other reported timing included within 3 months of first probable or definite TIA.^[21]^ On the other hand, 10 out of 16 studies have reported involvement of professionals: physiotherapists (n = 6: physiotherapists, kinesiologists, exercise therapists), nurses (n = 3), doctors (n = 2: physicians, cardiologists, neurologists), psychologists (n = 2: psychologists, neuropsychologists), pharmacists (n = 2), occupational therapists (n = 1) dieticians (n = 1), and nutritionists (n = 1).

## 4. Outcomes measures

The intervention programs reviewed in the selected studies did not focus on stroke literacy or knowledge outcomes. However, 7 articles^[21–23,26,28,31,32]^ examined blood pressure using measures like Systolic Blood Pressure (SBP), Diastolic Blood Pressure (DBP), and stroke risk score card. Only two studies measured blood glucose level utilizing glycated hemoglobin (HbA1c) and fasting glucose.^[21,26]^ Low-density lipoprotein-cholesterol (LDL-C) and total cholesterol levels were measured in 6 studies,^[21,23,28,29,31,32]^ while obesity was assessed in 6 studies^[21,23,28,29,32,34]^ in term of either abdominal obesity,^[34]^ body weight,^[29,32]^ body mass index (BMI) or waist circumference. Smoking behavior was analyzed in 3 studies (pharmacist-led case management, MoveIT, and MakeMyDay) in the form of smoking status^[28,32]^ and also as a key item in the stroke risk score card,^[23]^ with alcohol consumption behavior measured in the mHealth platform.^[23]^ Medication adherence, on the other hand, was accessed through reported medication adherence in 3 studies (pharmacist-led case management, TEAM, and clinical pharmacist intervention).^[26–28]^ Lifestyle modification programs like S + SLAM-TIA, MoveIT, and MakeMyDay measured exercise participation through adherence and satisfaction, PASE questionnaire and stroke risk scorecard,^[22,23,32]^ while cardiorespiratory fitness programs like PaP, PREVENT, Cardiac Rehabilitation (CR) Intervention, and MoveIT used measures like moderate-vigorous physical activity (MVPA), 6-minute walking distance (6MWD), maximal oxygen consumption (VO2Max), and resting heart rate (RHR).^[21,24,30,32,34]^

We also found 4 studies which examined the impact of programs on fatigue, cognitive impairments, mental health, weakness, balance, and quality of life. Fatigue was measured using the Fatigue Assessment Scale (FAS)^[21]^ and Fatigue Severity Scale^[32]^ assessments in the PREVENT and MoveIT programs. Cognitive impairment was assessed with Montreal Cognitive Assessment in the MoveIT RCT programs,^[32]^ while Mini-Mental State Examination, Digit Span, Clock Drawing, Trail Making, and FAS oral-verbal fluency (FAS-OVF) assessments were used in the UC CCR program.^[33]^ Mental health was primarily measured with Hospital Anxiety and Depression Scale^[21,32,33]^ and Center for Epidemiological Studies-Depression Scale.^[34]^ Finally, STROKEWALK in one study evaluated lower body strength through the 5-time chair stand test.^[30]^

## 5. Discussion

Health literacy among patients is a relevant topic worth discussing in relation to the need for information among post-TIA individuals. “Health literacy” was defined by the Centers for Disease Control and Prevention (CDC)^[35]^ as the degree to which individuals have the ability to find, understand, and use information and services to inform health-related decisions and actions for themselves and others. In the United States, there were more than 43 million people with inadequate health literacy,^[36]^ while in Malaysia, the overall health literacy status was documented as being at a lower sufficiency level, with limited health literacy level accounting for 32.3% in disease prevention, 27.9% in healthcare, and 26.6% in health promotion.^[37]^ The prevalent population with limited health literacy were respondents with older age (68%), lower education level (64.8%), and lower household income (49.5%).^[37]^ TIA incidence rate was reported at 18.8 to 28.6 in subjects aged 45 to 64 years and steeply increased from 71 to 141, peaking in subjects aged ≥85 years (229.2).^[38]^ It is clear that the need for information is important to be included in decisions or actions that may help reduce subsequent stroke risks and improve quality of life, especially for the older TIA patients who are from a lower health literacy category and may possibly overlap other factors such as low education and low household income. A qualitative study on TIA follow-up care experience has also highlighted the need for information, specifically the need to know about the diagnosis of TIA and subsequent stroke risks.^[13]^ However, when we look into the 16 selected studies, only 6 studies contained information sharing as a part of the program.^[19–24]^ None of these studies has highlighted the satisfaction or perceptions of patients or any outcomes of this supplementary program. Thus, the actual impact is not known. Due to the limited number of studies that have included the need for information as part of the care program, we could not confirm to what extent the current practice has followed.

A study reported that secondary care clinicians recognized it is not ideal to deliver information at the time of TIA diagnosis, and most general practitioners admitted not repeating information or checking patients’ understanding due to time constraints or assumptions this would be done in secondary care, leaving patients largely felt abandoned and alone post-discharge.^[13]^ A secondary stroke prevention program is a good platform to provide and reinforce an information that patients need to manage the risks of subsequent strokes. Anyhow, our review findings show that educating and reinforming patients about their diagnosis and subsequent stroke risk is not commonly practised. This could be possibly due to the easily accessible information through mobile internet usage, which has been sped up post-COVID pandemic.^[39]^ It may have reduced the prevalence of the need to depend on a healthcare provider for stroke information; however, the actual needs by 2023 remains unclear. Considering the gap of a few years from the stated needs in terms of information on diagnosis and stroke risk stated in 2019, there is a need to discover the experiences of TIA patients following the current secondary stroke prevention program, to what extent the education or information sharing has been executed in the current post-TIA care, and how prevalent is the need for information in today’s practice.

As for the content of stroke prevention program, all 16 studies documented relevant activities. Common remedies included targeting modifiable risk factors by controlling stroke risk factors and lifestyle modifications, which often span physical activity, diet, smoking cessation, and alcohol consumption. In this review, we found that most of the interventions involved were in the form of a mixed regimen, with a combination of exercises and lifestyle modification, or education, being the most common. Across all the interventions from the 16 studies, the interventions that were commonly used were providing education, suggested or prescribed exercise, counseling services, and a smoking cessation program. Among all, physical activity or exercise was one of the most used and suggested intervention by the rehabilitation team.^[20–22,24,29–34]^ Exercise is known for being safe and for its ability to bring positive benefits in stroke prevention.^[40–42]^

Recorded rehabilitation programs in this review were S + SLAM-TIA, PREVENT, cardiac rehabilitation, aerobic training, strength training, home-based exercise, SMS-directed training, and PaP. Our findings on the reported benefits from exercises starting from moderate intensity (RPE 12–13 or 60% intensity) were consistent with the existing literature, specifically in the domains of composite mobility, physical capacity, fatigue, health-related quality of life (HRQoL), neurologic functions, cardiovascular health, and depression. However, there is a statistic from the year of 2011 to be considered: 50% of people who start an exercise program will drop out within 6 months.^[43]^ Further, a past study also reported that significant predictors of dropout after 1 year were low education, low grip strength, lower cardiorespiratory fitness, low PA level, and randomization to supervised exercise.^[44]^ The same predictors of dropout were still significant after 3 years, with reduced memory status as an additional predictor.^[44]^ These factors were the results of dropouts after 6 months, which in turn strengthen the vicious cycle of physical inactivity, which then leads to failure in the stroke prevention exercise program. Nonetheless, the long-term impact of these programs, specifically the long-term adherence to the exercise program, which will, in turn, trigger a positive cycle in stroke risk prevention, is not known due to the relatively short period of the program (12 weeks).

To address the adherence issue, a program comprising aerobic and resistance exercises ≥ 2 times per week, supervised by a health professional (supplemented with a home program) over at least 24 weeks was suggested.^[45]^ In addition, group exercises that enhance social connectedness can also be considered in designing the exercise program. The design of group exercise has also been proven in the same study,^[45]^ reporting that the patients value the accompanying activities, which may change the patients’ physical activity behavior. Aside from that, themes encompassing participant perceptions of benefits, empowering or energizing effects, the instructor, and individual behavior can also be considered in designing the exercise program.^[46]^ In spite of the stated benefits of stroke risk stratification through exercise, our review found limited evidence with regard to cognitive function, anxiety and body composition despite the existence of confirming studies. Nonetheless, exercise is still one of the most abundantly used interventions post-TIA globally, aiming to build patients’ fitness and reduce stroke risk by lowering cardiovascular risks.

Lifestyle modification has been identified as an essential component of risk factor management that ought to be included as part of a comprehensive post-stroke rehabilitation.^[47]^ Medical adherence is one of the aspects of lifestyle modification being emphasized in the studies, with pharmacists specifically reviewing the medications and any adherence-related issues. Motivational interviewing is also used to further strengthening adherence; however, the approach has not yielded any significant outcomes, possibly due to the existing high adherence among the participants. Aside from that, McAlister et al^[28]^ have perhaps shown that medical adherence in the form of lifestyle advice or education is sufficient to work well with the targeted stroke risk factors. Besides, there are 3 studies which used counseling as one of their interventions. In the study conducted by Prior et al and Kamm et al., they provided dietary or nutritional counseling and psychological coping services.^[20,33]^ While the counseling intervention provided by Irewall et al^[31]^ was carried out through the follow-up method to provide lifestyle counseling, information about stroke, lifestyle changes including diet, alcohol, tobacco use, stress, and sleep were some of the common contents being touched on. Though not specified, all 3 studies involving counseling reported positive outcomes such as comparable blood pressure improvements, decline in vascular risk factors, and reduction in depression. In other studies, lifestyle counseling interventions were also shown to be successful in reducing the weight and alcohol consumption of post-stroke patients while also increasing variables that are linked to a healthy lifestyle.^[48]^

We also noted 4 studies that incorporated the patient-centered approach, where communications were done with patients to identify their goals and choose their desired interventions. However, adherence to physical activities was still low and was reported to be “hard to sustain.”^[23,32]^ Nonetheless, other benefits, such as increased awareness of risk factors, sustainable dietary goals, and improved cardiovascular health were noted.

The current secondary stroke prevention program has stepped ahead by utilizing technologies such as gadgets and mobile applications. In the study conducted by Vahlberg et al, telephone was used to deliver instructional text messages via SMS for 3 months daily, giving instructions on how to exercise to increase walking performance and improve lower body strength.^[30]^ Improvements in composite mobility, such as walking performance and strength, were noted. However, the study has not looked into the long-term feasibility and practicability of such approach. As for lifestyle modification, the Brain-Fit App was used in the studies conducted by Heron et al,^[19]^ while the mHealth platform was used by Patomella et al.^[23]^ In both applications, they shared a similar function, which is goal setting. This is crucial, as patient involvement in goal setting will increase their motivation to participate in the rehabilitation process, lead to greater achievement of their goals, and increase their satisfaction level.^[49]^ Involving patients in goal-setting will also help health professionals plan the most meaningful and suitable intervention for the patients.^[50]^ The Brain-Fit App provided information about TIA, suggestions on physical activity, a healthy diet, fatigue management, and goal setting,^[19]^ while the mHealth platform was used to monitor patient’s progress towards their goals, encourage them to participate in meaningful EEA, and facilitated their self-reflection on lifestyle habits.^[23]^ This model is similar to the current models of mobile applications designed for post-stroke participants, which mainly cover 3 domains: rehabilitation, education, and self-care.^[51]^ In another study, TEAM program was used^[26]^ in which the calls between TIA patients and nurse educators reinforced content from the group sessions, served as a behavioral model, provided social support, and facilitated linkage with other care providers like stroke nurses.^[26]^ These interventions adopted the model to enhance patients’ motivation, and all were shown to be feasible or acceptable and have increased patients’ awareness of the risk factors. The advancement of technologies has enhance the potential advantage of supplementing patients with the ability to modify their lifestyle through an online approach, and this may be incorporated as part of a care program complementing face-to-face exercises in future studies.^[51]^

In contrast to the vast body of research in stroke prevention, there is indeed a huge gap in the need for holistic care covering TIA patients’ residual impairments and the impacts of these impairments and the care programs on their quality of life. Residual symptoms post-TIA have been extensively reported since the last decade, primarily impacting patients’ quality of life,^[8]^ specifically cognitive, motor, mood, and sleep order. In our review, we only managed to identify 4 studies that looked into the components of holistic care. To overcome residual impairments, Heron’s Brain Fit App has inserted management tips for fatigue,^[19]^ although the effectiveness remains unknown. Fatigue was assessed in other exercise interventions, using FAS and FASS, showing the profound effect of aerobic exercise, strength training, and home-based exercises on this impairment.^[32]^ Specifically for cognitive impairment, Deijle’s MoveIT program has, however, shown limited evidence of effectiveness in improving Montreal Cognitive Assessment score.^[32]^ Similar to Prior’s findings, out of all cognitive measures utilized, only FAS-OVF yielded significant improvements.^[33]^ Depression was also addressed in a prospective cohort study by Prior et al that reported potential benefits from CCR psychological interventions on depression. However, the detailed intervention was not reported.^[33]^ Interestingly, Kamm et al included a defined session to address the specific problems of patients with stroke, including endurance training, fine motor skills, coordination, balance training, mobilization, or weight training which conducted by a physiotherapist specializing in neurorehabilitation.^[20]^ Despite being dedicated to stroke patients, the structure of the program may be modified to suit TIA patients’ needs. Secondary stroke prevention program post-TIA plays a vital role in supporting patients, and opportunities must be taken to respond to patients’ concerns that have significant implications for reducing stroke recurrence risk by 80%.^[52,53]^

The guideline by Kernan et al^[25]^ was comprehensive, covering recommendations for each subtype and comorbidity as well as the evidence level of each suggested intervention. However, the need for information and treatment for residual impairments was not highlighted. By the time this review was completed, studies specifically investigating interventions for residual impairments or the impacts of residual impairments on patients’ lives were found lacking.

Currently, the available evidence still focuses on stroke prevention, and our findings are in line with a recent study,^[14]^ which found that there are indeed no guidelines that specifically address how best to support patients following TIA or minor strokes to resume usual daily activities. Future studies involving interventions on residual impairments and TIA patients’ lives are warranted. Possibly, assessments pertaining to stroke knowledge, health states, cardiovascular risk factors and residual impairments could be suggested in future studies, in view to explore the effectiveness of the care program in addressing each needs domain of the post-TIA individuals.

### 5.1. Strength and limitation of this review

To the best of our knowledge, this is the first scoping review looking into the available care programs specifically targeting the needs of 3 domains: The need for information, Stroke prevention, and Holistic care. In this scoping review, the aim was to map the evidence of existing care programs or guidelines to the 3 domains of needs. Thus, all study types, excluding reviews, were included, and this has provided us with a broader spectrum of evidence, allowing us to map each study to the relevant needs that they are addressing, demonstrating saturations and lacks in each domain. Subsequently, a foundation for future research initiatives is founded by providing insights into the current state of knowledge and outlining potential study directions. Further, in addition to the components of the care program, timing and professionals involved are also noted in this scoping review, providing a multi-dimensional view into the existing care programs, which may inform the potential structure of care programs dedicated to post-TIA patients.

Nonetheless, this scoping review is subjected to several limitations. First, the research searches were restricted to only 4 databases deemed most pertinent to the review scope. While these databases were chosen based on their relevance to the topic at hand, it is essential to acknowledge that other databases might have contained relevant studies that were not included in our search. Moreover, the inclusion criteria focused solely on the studies written in English over the past 10 years to manage the dataset effectively. This timeframe was chosen to ensure the inclusion of recent research, while the language was chosen to ensure thorough review and accurate analysis. As a consequence, there is a possibility that our evaluation method may have led to missed research papers. Third, due to the large pool of articles obtained initially, multiple screenings have been done via an advanced search of keywords. This may lead to the potential loss of relevant studies compared to manual checking.

## 6. Conclusion

This scoping review explored the evidence of existing care programs for patients following TIA. We can conclude that currently, available care is more oriented toward secondary stroke prevention with common interventions such as physical activity, lifestyle modification given through a behavior model and yielding positive outcomes. However, limited evidence of practice is available in the context of information provisions, and the gap is especially huge when it comes to the context of post-TIA residual impairments management and their impacts on patients’ lives. Nonetheless, this scoping review identified samples of care program components that could be incorporated in developing a multi-component care module to fulfill the multiple needs of post-TIA individuals.

## Acknowledgments

The authors thank the Ministry of Higher Education, Malaysia for this study funding (grant code FRGS/1/2023/SS09/UKM/02/1).

## Author contributions

**Conceptualization:** Nor Azlin Mohd Nordin, Aznida Firzah Abdul Aziz.

**Funding acquisition:** Nor Azlin Mohd Nordin.

**Supervision:** Nor Azlin Mohd Nordin, Aznida Firzah Abdul Aziz.

**Writing – original draft:** Wei Sheng Ho.

**Writing – review & editing:** Wei Sheng Ho, Nor Azlin Mohd Nordin.

## References

[1] Easton JD Saver JL Albers GW.; American Heart Association. Definition and evaluation of transient ischemic attack. Stroke. 2009;40:2276–93. 19423857 10.1161/STROKEAHA.108.192218

[2] Panuganti KK Tadi P Lui F Transient ischemic attack. In: StatPearls. Updated July 17, 2023. StatPearls Publishing; 2025. https://www.ncbi.nlm.nih.gov/sites/books/NBK459143/. 29083778

[3] Tan KS Venketasubramanian N. Stroke burden in Malaysia. Cerebrovasc Dis Extra. 2022;12:58–62. 35325896 10.1159/000524271 PMC9149343

[4] Spurgeon L Humphreys G James G Sackley C. A Q-methodology study of patients’ subjective experiences of TIA. Stroke Res Treat. 2012;2012:1–10. 10.1155/2012/486261 PMC3398653 22848864

[5] Moran GM Fletcher B Feltham MG Calvert M Sackley C Marshall T. Fatigue, psychological and cognitive impairment following transient ischaemic attack and minor stroke: a systematic review. Eur J Neurol. 2014;21:1258–67. 24861479 10.1111/ene.12469

[6] Batchelor FA Williams SB Wijeratne T Said CM Petty S. Balance and gait impairment in transient ischemic attack and minor stroke. J Stroke Cerebrovasc Dis. 2015;24:2291–7. 26227322 10.1016/j.jstrokecerebrovasdis.2015.06.014

[7] Li N Li J Gao T Wang D Du Y Zhao X. Gait and balance disorder in patients with transient ischemic attack or minor stroke. Neuropsychiatr Dis Treat. 2021;17:305–14. 33568910 10.2147/NDT.S289158 PMC7868302

[8] Lodha N Harrell J Eisenschenk S Christou EA. Motor impairments in transient ischemic attack increase the odds of a subsequent stroke: a meta-analysis. Front Neurol. 2017;8:243. 28638365 10.3389/fneur.2017.00243 PMC5461338

[9] Hasan F Gordon C Wu D. Dynamic prevalence of sleep disorders following stroke or transient ischemic attack. Stroke. 2021;52:655–63. 33406871 10.1161/STROKEAHA.120.029847

[10] Coutts SB Modi J Patel SK. What causes disability after transient ischemic attack and minor stroke? Stroke. 2012;43:3018–22. 22984013 10.1161/STROKEAHA.112.665141

[11] Moran GM Fletcher B Calvert M Feltham MG Sackley C Marshall T. A systematic review investigating fatigue, psychological and cognitive impairment following Tia and minor stroke: protocol paper. Syst Rev. 2013;2:72. 24011357 10.1186/2046-4053-2-72 PMC3846122

[12] Turner GM Backman R McMullan C Mathers J Marshall T Calvert M. Establishing research priorities relating to the long-term impact of Tia and minor stroke through stakeholder-centred consensus. Res Involv Engagem. 2018;4:2. 29416879 10.1186/s40900-018-0089-z PMC5784709

[13] Turner GM McMullan C Atkins L Foy R Mant J Calvert M. Tia and minor stroke: a qualitative study of long-term impact and experiences of follow-up care. BMC Fam Pract. 2019;20:10. 31847828 10.1186/s12875-019-1057-x PMC6918619

[14] Hede Ebbesen B Modrau B Kontou E. Lasting impairments following transient ischemic attack and minor stroke: a systematic review protocol. Front Neurol. 2023;14:1177309. 37251235 10.3389/fneur.2023.1177309 PMC10213239

[15] Crow J Savage M Gardner L. What follow-up interventions, programmes and pathways exist for minor stroke survivors after discharge from the acute setting? A scoping review. BMJ Open. 2023;13:e070323. 10.1136/bmjopen-2022-070323 PMC10277077 37311634

[16] Arksey H O’Malley L. Scoping studies: towards a methodological framework. Int J Soc Res Methodol. 2005;8:19–32.

[17] Tricco AC Lillie E Zarin W. Prisma extension for scoping reviews (PRISMA-SCR): checklist and explanation. Ann Intern Med. 2018;169:467–73. 30178033 10.7326/M18-0850

[18] Qi X Yang M Ren W. Find duplicates among the pubmed, EMBASE, and Cochrane Library Databases in systematic review. PLoS One. 2013;8:e070323. 10.1371/journal.pone.0071838 PMC3748039 23977157

[19] Heron N O’Connor SR Kee F. Development of a digital lifestyle modification intervention for use after transient ischaemic attack or minor stroke: a person-based approach. Int J Environ Res Public Health. 2021;18:4861. 34063298 10.3390/ijerph18094861 PMC8124154

[20] Kamm CP Schmid J-P Müri RM Mattle HP Eser P Saner H. Interdisciplinary cardiovascular and neurologic outpatient rehabilitation in patients surviving transient ischemic attack or stroke with minor or no residual deficits. Arch Phys Med Rehabil. 2014;95:656–62. 24184308 10.1016/j.apmr.2013.10.013

[21] MacKay-Lyons M Gubitz G Phillips S. Program of Rehabilitative Exercise and education to avert vascular events after non-disabling stroke or transient ischemic attack (prevent trial): a randomized controlled trial. Neurorehabil Neural Repair. 2021;36:119–30. 34788569 10.1177/15459683211060345 PMC9066689

[22] Sammut M Haracz K English C. Participants’ perspective of engaging in a gym-based Health Service delivered secondary stroke prevention program after TIA or mild stroke. Int J Environ Res Public Health. 2021;18:11448. 34769964 10.3390/ijerph182111448 PMC8583419

[23] Patomella A-H Farias L Eriksson C Guidetti S Asaba E. Engagement in everyday activities for prevention of stroke: feasibility of an mhealth-supported program for people with tia. Healthcare (Basel). 2021;9:968. 34442105 10.3390/healthcare9080968 PMC8393226

[24] Morén C Welmer A-K Hagströmer M Karlsson E Sommerfeld DK. The effects of “Physical activity on prescription” in persons with transient ischemic attack: a randomized controlled study. J Neurol Phys Ther. 2016;40:176–83. 27176943 10.1097/NPT.0000000000000134

[25] Kernan WN Ovbiagele B Black HR.; American Heart Association Stroke Council, Council on Cardiovascular and Stroke Nursing, Council on Clinical Cardiology, and Council on Peripheral Vascular Disease. Guidelines for the prevention of stroke in patients with stroke and transient ischemic attack: a guideline for healthcare professionals from the American Heart Association/American Stroke Association [published correction appears in Stroke. 2015 Feb;46(2):e54. doi: 10.1161/STR.0000000000000059]. Stroke. 2014;45:2160–236. 24788967 10.1161/STR.0000000000000024

[26] Sajatovic M Tatsuoka C Welter E. A targeted self-management approach for reducing stroke risk factors in African American men who have had a stroke or transient ischemic attack. Am J Health Promot. 2017;32:282–93. 28530142 10.1177/0890117117695218 PMC6241515

[27] Hedegaard U Kjeldsen LJ Pottegård A Bak S Hallas J. Multifaceted intervention including motivational interviewing to support medication adherence after stroke/transient ischemic attack: a randomized trial. Cerebrovasc Dis Extra. 2014;4:221–34. 25598772 10.1159/000369380 PMC4296247

[28] McAlister FA Majumdar SR Padwal RS. Case management for blood pressure and lipid level control after minor stroke: prevention randomized controlled trial. CMAJ. 2014;186:577–84. 24733770 10.1503/cmaj.140053 PMC4016053

[29] Vahlberg BM Lundström E Eriksson S Holmbäck U Cederholm T. Potential effects on cardiometabolic risk factors and body composition by Short Message Service (SMS)-guided training after recent minor stroke or transient ischaemic attack: post hoc analyses of the STROKEWALK randomised controlled trial. BMJ Open. 2021;11:e054851. 10.1136/bmjopen-2021-054851 PMC8524288 34663672

[30] Vahlberg B Lundström E Eriksson S Holmbäck U Cederholm T. Effects on walking performance and lower body strength by short message service guided training after stroke or transient ischemic attack (the STROKEWALK study): a randomized controlled trial. Clin Rehabil. 2020;35:276–87. 32942914 10.1177/0269215520954346 PMC7874373

[31] Irewall A-L Ögren J Bergström L Laurell K Söderström L Mooe T. Nurse-led, telephone-based secondary preventive follow-up benefits stroke/TIA patients with low education: a randomized controlled trial sub-study. Trials. 2019;20:52. 30646948 10.1186/s13063-018-3131-4 PMC6334622

[32] Deijle IA Hemmes R Boss HM. Effect of an exercise intervention on global cognition after transient ischemic attack or minor stroke: the MOVEIT randomized controlled trial. BMC Neurol. 2022;22:289. 35927622 10.1186/s12883-022-02805-z PMC9351151

[33] Prior PL Hachinski V Chan R. Comprehensive cardiac rehabilitation for secondary prevention after transient ischemic attack or mild stroke. J Cardiopulm Rehabil Prev. 2017;37:428–36. 28727668 10.1097/HCR.0000000000000274

[34] Marzolini S Danells C Oh PI Jagroop D Brooks D. Feasibility and effects of cardiac rehabilitation for individuals after transient ischemic attack. J Stroke Cerebrovasc Dis. 2016;25:2453–63. 27425176 10.1016/j.jstrokecerebrovasdis.2016.06.018

[35] What is health literacy? Centers for Disease Control and Prevention. https://www.cdc.gov/health-literacy/php/about/index.html. Accessed May 5, 2024.

[36] Shahid R Shoker M Chu LM Frehlick R Ward H Pahwa P. Impact of low health literacy on patients’ health outcomes: a multicenter cohort study. BMC Health Serv Res. 2022;22:1148. 36096793 10.1186/s12913-022-08527-9 PMC9465902

[37] Jaafar N Perialathan K Krishnan M. Malaysian health literacy: scorecard performance from a national survey. Int J Environ Res Public Health. 2021;18:5813. 34071455 10.3390/ijerph18115813 PMC8197907

[38] Degan D Ornello R Tiseo C. Epidemiology of transient ischemic attacks using time- or tissue-based definitions. Stroke. 2017;48:530–6. 28143922 10.1161/STROKEAHA.116.015417

[39] Jonnatan L Seaton CL Rush KL Li EPH Hasan K. Mobile device usage before and during the COVID-19 pandemic among rural and urban adults. Int J Environ Res Public Health. 2022;19:8231. 35886082 10.3390/ijerph19148231 PMC9315523

[40] Faulkner J Stoner L Lambrick D. Physical activity and exercise engagement in patients diagnosed with transient ischemic attack and mild/non-disabling stroke: a commentary on current perspectives. Rehabil Process Outcome. 2014;3:19–24.

[41] Thurston C Bezuidenhout L Humphries S. Mobile Health to promote physical activity in people post stroke or transient ischemic attack – study protocol for a feasibility randomised controlled trial. BMC Neurol. 2023;23:124. 36978045 10.1186/s12883-023-03163-0 PMC10043533

[42] Boss HM Van Schaik SM Deijle IA. Safety and feasibility of post-stroke care and exercise after minor ischemic stroke or transient ischemic attack: motives & moveit. NeuroRehabilitation. 2014;34:401–7. 24473242 10.3233/NRE-141049

[43] Linke SE Gallo LC Norman GJ. Attrition and adherence rates of sustained vs. intermittent exercise interventions. Ann Behav Med. 2011;42:197–209. 21604068 10.1007/s12160-011-9279-8 PMC3181282

[44] Viken H Reitlo LS Zisko N. Predictors of dropout in exercise trials in older adults: the generation 100 study. Med Sci Sports Exerc. 2019;51:49–55. 30113524 10.1249/MSS.0000000000001742

[45] Sammut M Fini N Haracz K Nilsson M English C Janssen H. Increasing time spent engaging in moderate-to-vigorous physical activity by community-dwelling adults following a transient ischemic attack or non-disabling stroke: a systematic review. Disabil Rehabil. 2020;44:337–52. 32478574 10.1080/09638288.2020.1768599

[46] Farrance C Tsofliou F Clark C. Adherence to community based group exercise interventions for older people: a mixed-methods systematic review. Prev Med. 2016;87:155–66. 26921655 10.1016/j.ypmed.2016.02.037

[47] Bailey RR. Lifestyle modification for secondary stroke prevention. Am J Lifestyle Med. 2016;12:140–7. 30202386 10.1177/1559827616633683 PMC6124986

[48] Oikarinen A Engblom J Paukkonen L Kääriäinen M Kaakinen P Kähkönen O. Effects of a lifestyle counselling intervention on adherence to lifestyle changes 7 years after stroke – a quasi‐experimental study. Scand J Caring Sci. 2022;37:163–72. 35766254 10.1111/scs.13101

[49] Preede L Soberg HL Dalen H. Rehabilitation goals and effects of goal achievement on outcome following an adapted physical activity-based rehabilitation intervention. Patient Prefer Adherence. 2021;15:1545–55. 34276210 10.2147/PPA.S311966 PMC8277449

[50] Lopez L Holt L Jackson K. Goal setting in rehabilitation. Physiopedia. https://www.physio-pedia.com/Goal_Setting_in_Rehabilitation. Accessed April 5, 2024.

[51] Cao W Kadir AA Tang W Wang J Yuan J Hassan II. Effectiveness of mobile application interventions for stroke survivors: systematic review and meta-analysis. BMC Med Inform Decis Mak. 2024;24:6. 38167316 10.1186/s12911-023-02391-1 PMC10763083

[52] Doogue R McCann D Fitzgerald N Murphy AW Glynn LG Hayes P. Blood pressure control in patients with a previous stroke/transient ischaemic attack in primary care in Ireland: a Cross Sectional Study. BMC Fam Pract. 2020;21:139. 32650725 10.1186/s12875-020-01211-z PMC7353812

[53] Norrving B Barrick J Davalos A. Action plan for stroke in Europe 2018–2030. Eur Stroke J. 2018;3:309–36. 31236480 10.1177/2396987318808719 PMC6571507

